# Cross-sectional correlates and indirect associations linking parental warmth to scores on a study-specific gaming-related symptom index in senior-high-school and university students

**DOI:** 10.3389/fpsyt.2026.1928745

**Published:** 2026-09-29

**Authors:** Ruoyu Zhou, Wenjie Yang, Haotian Gao, Ruifeng Zhao

**Affiliations:** 1 School of Future Education, Qingdao Hengxing University of Science and Technology, Qingdao, Shandong, China; 2 School of Education, Yunnan University, Kunming, Yunnan, China; 3 Doctoral Program in Public Health, University of Tsukuba, Tsukuba, Ibaraki, Japan

**Keywords:** emotional distress, escape motivation, gaming-related symptom index, indirect association, parental warmth, self-control

## Abstract

**Introduction:**

Game escape motivation has been associated with internet gaming disorder (IGD) in previous research, but its role in family-linked associations remains unclear.

**Methods:**

In a cross-sectional survey of Chinese senior-high-school students (n = 145) and university students (n = 285), we estimated parallel indirect-association models linking parental warmth to scores on an 11-item study-specific gaming-related symptom index through escape motivation, self-control, and emotional distress. The index was substantially adapted from the Gaming Disorder Scale for Adolescents (GADIS-A) and has not undergone independent validation.

**Results:**

Emotional distress statistically accounted for part of the association between parental warmth and index scores in both groups, and an indirect association through self-control was detected in the senior-high-school group. Escape motivation showed positive adjusted coefficients, but no indirect association through escape motivation was detected in either group.

**Discussion:**

These findings concern scores on an unvalidated study-specific measure, not established IGD symptom severity. The cross-sectional design does not establish temporal mechanisms or developmental differences.

## 1 Introduction

Internet gaming disorder (IGD)—a pattern of persistent, impairing gaming recognized as a condition for further study in the DSM-5 (1) and included, as gaming disorder, in the ICD-11 (2)—has drawn sustained clinical and public-health attention. Its pooled prevalence is generally in the low single digits, with substantial between-study heterogeneity (3). However, frequent engagement should not be equated with clinical disorder; population evidence indicates that functional impairment is concentrated in a much smaller subgroup than high gaming involvement itself (4). Escape motivation is among the most consistent correlates of IGD severity (5, 6). The present study addresses a narrower question: whether escape motivation statistically accounts for an association between a more distal family-context variable and scores on a study-specific gaming-related symptom index when other candidate intervening variables are considered in parallel.

### 1.1 Escape motivation as a correlate of IGD

The compensatory framework proposes that gaming may become problematic when it is used to cope with distress, unmet needs, or aversive states (6). The I-PACE model likewise situates affective responses, coping, and executive control within interacting processes rather than a single-cause chain (7). Empirically, escape or mood-regulation motives are among the strongest motivational correlates of IGD across samples and instruments (5, 8), and prior work has described escape as a correlate or potential mediator of problematic gaming (9). The present study distinguishes the adjusted association of escape motivation with index scores from its statistical role in the association between parental warmth and index scores.

### 1.2 Adjusted association and statistical indirect association are different questions

An adjusted association with an outcome score and a statistical indirect association answer different questions (10, 11). We therefore examined both the adjusted association between escape motivation and index scores and the indirect association linking parental warmth to index scores through escape motivation.

### 1.3 Parental warmth and candidate intervening variables

Parental emotional warmth and supportive parenting are associated with lower IGD, particularly in younger samples (12, 13). A two-wave study of Chinese adolescents also linked parental involvement to later IGD through future-oriented cognition, illustrating that family effects may operate through several candidate processes rather than one fixed pathway (14). Self-determination theory offers one possible account: supportive family relationships may be associated with autonomous regulation, basic-need satisfaction, and lower negative affect (15). Emotional distress is also a stable correlate of IGD (16), whereas self-control is negatively associated with problematic Internet use and IGD-related outcomes (17, 18). We therefore examined emotional distress, self-control, and escape motivation in parallel in relation to index scores. The resulting indirect associations remain compatible with reverse or reciprocal relations.

### 1.4 Comparison of two educational-stage groups

Senior-high-school and university students differ not only in age (19) but also in educational setting, recruitment context, and other characteristics. We therefore estimated the models separately by group and avoided interpreting group-specific patterns as direct evidence of developmental differences.

### 1.5 The present study

Using Chinese senior-high-school and university student samples, we estimated the association between parental warmth and scores on a study-specific gaming-related symptom index and decomposed it into parallel indirect associations through escape motivation, self-control, and emotional distress. Analyses were not preregistered and are interpreted as theory-guided rather than confirmatory. We asked: (RQ1) Was an indirect association through escape motivation statistically detectable in either group? We expected (H1) an indirect association through emotional distress in both groups and (H2) an inverse indirect association through self-control in the senior-high-school group. 

## 2 Materials and methods

### 2.1 Participants, recruitment, and data screening

Participants were Chinese students recruited online through three senior high schools and four universities in Shandong Province in 2026; participation was voluntary and unpaid. Institution names and institution-level identifiers were not retained in the deidentified dataset in accordance with the confidentiality arrangements. Of 551 returned questionnaires, 38 were excluded for completion times shorter than 120 s, 80 for zero within-scale variance across all 13 items of the Brief Self-Control Scale, and 3 because age or grade was outside the target range or group assignment was not possible. The 80 zero-variance cases also showed a broader response-quality pattern, including shorter completion times, longer identical-response runs, fewer response categories, and lower within-person response variance than retained cases (**Supplementary Table S6**). The final analytic sample was N = 430, with no missing data on the primary variables. Participant flow and exclusions are summarized in **Table 1**.

**Table 1:** Participant flow.

Step	Excluded n	Retained n
Returned questionnaires	—	551
Completion time < 120 s	38	513
Zero variance on self-control scale	80	433
Age/grade ineligible or group unassignable	3	430
Final senior-high-school group	—	145
Final university group	—	285

The final groups comprised 145 senior-high-school students (76 female, 69 male; M age = 16.10, SD = 0.86) and 285 university students (195 female, 90 male; M age = 20.12, SD = 1.40). Written informed consent was obtained from university participants and from the parents or legal guardians of minor participants. Minor participants additionally provided assent, and permission was obtained from the participating schools.

Gaming time was assessed with the item, ‘On average, how long do you play games each day?’ The six response options were: ‘I do not play,’ ‘less than 1 hour,’ ‘1–2 hours,’ ‘2–4 hours,’ ‘4–6 hours,’ and ‘6 hours or more.’ Respondents selecting ‘I do not play’ were not excluded on the basis of this response; daily gaming time was coded as the lowest ordinal category, and their recorded responses to the gaming-specific measures were retained rather than recoded to zero. In the final analytic sample, 33 senior-high-school students (22.8%) and 75 university students (26.3%) selected ‘I do not play.’ A sensitivity analysis excluding these respondents is reported in **Supplementary Table S3**.

### 2.2 Measures

Identical instruments were administered to both groups. Parental emotional warmth was measured with the 7-item warmth subscale of the Chinese short-form EMBU (s-EMBU-C) (20), using a 4-point response scale. Basic psychological need satisfaction was additionally measured with 12 items from the satisfaction subscale of Chen et al. (21). Because this scale indexes general need satisfaction rather than a family-specific construct, it was used only in robustness analyses. Escape motivation was measured with the 4-item escape subscale of the Motives for Online Gaming Questionnaire (MOGQ) (8), using the Chinese version (22). Self-control was measured with the 13-item Brief Self-Control Scale (17), with nine reverse-keyed items recoded so that higher scores indicated greater self-control.

The primary outcome was the mean score on an 11-item study-specific gaming-related symptom index, administered in Chinese and substantially adapted from the Gaming Disorder Scale for Adolescents (GADIS-A) (23, 24). The index involved substantive item reformulation and supplementation and has not undergone independent validation. It is not treated as an established measure of IGD symptom severity. Items were rated from 1 (never) to 5 (always), and their mean was analyzed as a continuous index score without diagnostic cut-offs or case classification. The adaptation rationale and administered item wording are reported in **Supplementary Section S2**; sample-specific measurement and scoring-sensitivity results are reported in **Supplementary Sections S7**, **S8**, and **S10**.

Emotional distress was measured with the PHQ-2 (25) and GAD-2 (26), recoded to the conventional 0–3 metric and averaged as a four-item general-distress index. The index is not interpreted as a diagnosis of depression or anxiety. A one-factor model fit the senior-high-school data well (CFI = 1.000, RMSEA = .000, SRMR = .009; loadings = .853–.938; α = .944, ω = .944). In the university group, loadings and reliability remained strong (.800–.905; α = .924, ω = .926), but fit was mixed (CFI = .985, RMSEA = .151, SRMR = .025); the elevated RMSEA should be interpreted cautiously because the model had only 2 degrees of freedom.

Observed-score internal consistencies (senior-high-school/university) were: parental warmth α = .95/.96; need satisfaction α = .97/.96; escape motivation α = .93/.93; self-control α = .89/.77; study-specific index α = .96/.95; and emotional distress α = .94/.92. Covariates were sex, age centered within group, and daily gaming time.

### 2.3 Analytic strategy and reporting

Within each group, a parallel multiple-intervening-variable model was estimated with parental warmth as the antecedent, escape motivation, self-control, and emotional distress entered simultaneously, the study-specific index score as the outcome, and sex (1 = male, 2 = female), group-centered age, and ordinal daily gaming duration as covariates. Continuous study variables were standardized across the full analytic sample before the group-specific models were fitted. The total association (c), direct association (c′), a paths, and adjusted b paths were estimated by ordinary least squares. Heteroscedasticity-consistent HC3 standard errors were used for coefficient tests. Indirect associations (a × b) were estimated separately within each group using 5,000 case-resampled percentile-bootstrap samples and 95% confidence intervals (27). Analyses were reproduced in Python 3.12.9 using pandas 2.2.3, NumPy 2.2.6, and SciPy 1.15.2. No primary analysis variable was missing. Because item-level metric invariance was not supported for self-control, self-control-related coefficients were not formally compared across groups.

Indirect effects are reported as statistical indirect associations, and non-significant estimates are described as not detected rather than absent (28).

A latent-variable SEM was used as a convergent analysis. Parental warmth, self-control, and the study-specific index were each represented by three balanced item parcels, with parceling treated as a pragmatic modeling choice rather than a substitute for item-level evidence (29); escape motivation and distress were represented by items. Indirect associations used 1,000 bootstrap resamples. Measurement invariance was assessed across groups, with |ΔCFI| ≤.01 as the primary criterion (30).

During revision, *post hoc* checks examined item-level diagnostics, associations with self-reported gaming duration, and alternative outcomes using the H1–H6 and H7–H11 domain means. These analyses used the unchanged final sample and covariates; estimation details, code, and results are provided in **Supplementary Section S10**.

## 3 Results

### 3.1 Descriptive statistics, correlations, and conditional associations

Observed-score means and correlations with the study-specific index score are shown in **Table 2**; the complete correlation matrices are reported in **Supplementary Table S5**. Parental warmth correlated strongly and negatively with the index score in the senior-high-school group (r = −.64) and weakly in the university group (r = −.16). Escape motivation was essentially unrelated to the index score at the zero-order level among senior-high-school students (r = .04) but moderately correlated among university students (r = .34). Self-control correlated negatively with the index score in both groups (r = −.58 and −.34), whereas emotional distress correlated positively (r = .42 and .39).

**Table 2:** Descriptive statistics, reliability, and zero-order correlations with scores on the study-specific gaming-related symptom index by educational group.

Variable	SHS M (SD)	SHS α	SHS r with index scores	Univ. M (SD)	Univ. α	Univ. r with index scores
Parental warmth	3.08 (0.82)	.95	−.64	3.20 (0.74)	.96	−.16
Need satisfaction	3.81 (0.98)	.97	−.70	4.02 (0.66)	.96	−.23
Escape motivation	2.97 (1.31)	.93	.04	2.74 (1.16)	.93	.34
Self-control	3.24 (0.84)	.89	−.58	3.03 (0.50)	.77	−.34
Emotional distress	1.36 (0.96)	.94	.42	0.82 (0.63)	.92	.39
Gaming-related index score	2.00 (1.04)	.96	—	1.80 (0.75)	.95	—

SHS, senior-high-school students (n = 145); Univ., university students (n = 285); M, mean; SD, standard deviation; α, Cronbach’s alpha; r, zero-order correlation with the study-specific gaming-related symptom index.

The senior-high-school escape-motivation coefficient changed from r = .04 at the zero-order level to a positive adjusted b path of .137 after parental warmth, self-control, distress, and covariates were included. Sequential models showed that the coefficient became positive mainly when self-control was controlled (escape motivation correlated positively with self-control, r = .385, whereas self-control correlated negatively with the index score, r = −.576), a classic suppression pattern. Collinearity was modest (maximum VIF = 2.32; escape-motivation VIF = 1.33). The coefficient was nominally significant with conventional standard errors (p = .028) but not with HC3 robust standard errors (p = .065). It is therefore not interpreted as a reliable independent correlate in this group. The university-group maximum VIF was 1.41.

### 3.2 Indirect associations linking parental warmth to index scores

The observed-score indirect-association estimates are presented in **Table 3**. In the senior-high-school group, parental warmth was associated with higher self-control (a = .592), and self-control was inversely associated with the index score after adjustment (b = −.322), producing an indirect association of −.191 (95% CI [−.306, −.096]). Parental warmth was associated with lower distress (a = −.555), and distress was positively associated with the index score (b = .251), producing an indirect association of −.139 (95% CI [−.216, −.075]). The specified model did not detect an indirect association through escape motivation (a × b = .020, 95% CI [−.010, .058]).

**Table 3:** Parallel indirect-association models with the study-specific index score as the outcome, by educational group (standardized coefficients; 5,000 bootstrap resamples).

Group	Indirect association via	a	b	a × b	95% CI	Result
Senior high school	Escape motivation	.145	.137	.020	[−.010, .058]	Not detected
Senior high school	Self-control	.592***	−.322***	−.191	[−.306, −.096]	Detected
Senior high school	Emotional distress	−.555***	.251***	−.139	[−.216, −.075]	Detected
University	Escape motivation	.081	.203***	.016	[−.009, .044]	Not detected
University	Self-control	.176***	−.076	−.013	[−.038, .008]	Not detected
University	Emotional distress	−.177***	.280***	−.050	[−.094, −.018]	Detected

Covariates were sex, group-centered age, and daily gaming time. Coefficient significance markers use HC3 robust standard errors; indirect-association inferences use percentile-bootstrap confidence intervals. ***p < .001.

In the university group, the indirect association through emotional distress was −.050 (95% CI [−.094, −.018]). The model did not detect indirect associations through self-control (−.013, 95% CI [−.038, .008]) or escape motivation (.016, 95% CI [−.009, .044]). Escape motivation nevertheless showed a positive adjusted association with index scores in this group (b = .203, p < .001).

Statistical detection in one group but not the other was not interpreted as evidence of a between-group difference. 

### 3.3 Sensitivity analyses

Using need satisfaction alone or a warmth-plus-need-satisfaction composite as the antecedent produced the same significance pattern as the primary models. Sensitivity analyses retaining the 79 otherwise eligible zero-variance cases are reported in **Supplementary Table S4**. Two small university-group indirect associations became statistically detectable. Because the zero-variance cases also showed converging indicators of low-quality responding (**Supplementary Table S6**), the screened sample was retained for the primary analyses.

A sensitivity analysis excluding participants who selected ‘I do not play’ is reported in **Supplementary Table S3**. The primary inferential pattern was unchanged.

In the additional scoring-sensitivity analyses, replacing the 11-item outcome with either domain mean preserved the pattern of confidence-interval exclusion for the indirect associations (**Supplementary Table S10c**). The full index was positively associated with self-reported gaming duration (Spearman ρ = .510 in senior-high-school students and .479 in university students; **Supplementary Table S10b**). These same-sample checks address the examined scoring choices, not diagnostic validity or equivalence with GADIS-A.

### 3.4 Measurement invariance and latent-variable analysis

The parcel-based five-factor configural model fit well (CFI = .988, RMSEA = .042, SRMR = .033). Full loading constraints yielded ΔCFI = −.005, although the χ² difference test was significant. Freeing one warmth parcel, one self-control parcel, and one distress item yielded partial metric invariance (ΔCFI = −.002); partial scalar invariance was also supported. Under these constraints, the indirect-association pattern was unchanged.

Item-level follow-up analyses qualified the parcel results. By the ΔCFI criterion, metric and scalar invariance were supported for parental warmth (metric ΔCFI = −.005; scalar ΔCFI = −.002) and distress (−.003 and <.001). For the two-factor study-specific gaming-related symptom index, the corresponding ΔCFI values were .001 and −.002, but these small changes do not resolve the imperfect underlying model fit, particularly in university students (CFI = .926, TLI = .906, RMSEA = .145, SRMR = .048; **Supplementary Sections S7, S8**). These results do not establish criterion or diagnostic validity, equivalence with GADIS-A, or unqualified cross-group comparability. The self-control item-level model showed poor configural fit (CFI = .679, RMSEA = .110), and metric invariance was not supported (ΔCFI = −.026). Accordingly, self-control associations were interpreted cautiously within groups, and their coefficient magnitudes were not formally compared across groups.

The latent-variable SEM fit acceptably by conventional criteria (31) in the senior-high-school group (CFI = .963, TLI = .955, RMSEA = .067) and the university group (CFI = .972, TLI = .965, RMSEA = .056). The indirect-association pattern matched the observed-score models (**Table 4**). The group-specific latent-variable models are shown in **Figure 1**. Because the senior-high-school sample was modest for the latent model and parcel construction involves researcher decisions, these results are treated as convergent rather than definitive evidence. 

**Table 4:** Latent-variable SEM indirect associations for the study-specific gaming-related index (1,000 bootstrap resamples).

Group	Indirect association via	a	b	a × b	95% CI	Result
Senior high school	Escape motivation	.160	.130*	.021	[−.009, .058]	Not detected
Senior high school	Self-control	.395***	−.502***	−.198	[−.332, −.096]	Detected
Senior high school	Emotional distress	−.572***	.242***	−.138	[−.227, −.071]	Detected
University	Escape motivation	.075	.206***	.015	[−.010, .043]	Not detected
University	Self-control	.107***	−.094	−.010	[−.035, .011]	Not detected
University	Emotional distress	−.170***	.310***	−.053	[−.100, −.018]	Detected

Coefficients are standardized. CI, confidence interval. *p < .05; ***p < .001.

**Figure 1:**
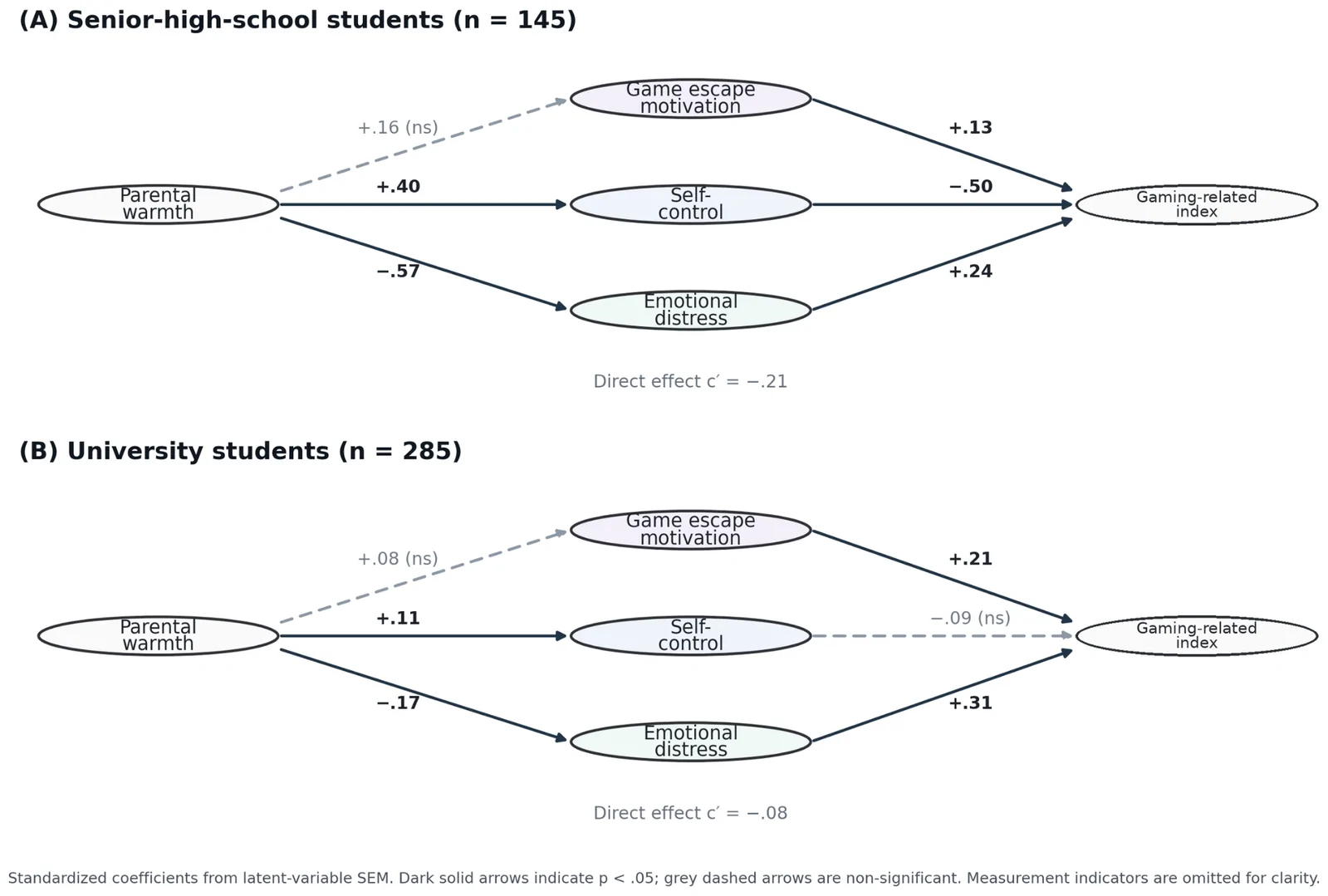
Latent-variable SEM by educational group. Solid paths indicate p <.05; dashed paths are non-significant. Measurement indicators are omitted for clarity. The outcome factor was represented by parcels of the 11 study-specific items and is not interpreted as a validated latent IGD construct.

### 3.5 Diagnostics, explained variance, and clustering

The senior-high-school models explained 8.5% of the variance in escape motivation, 49.6% in self-control, 29.1% in distress, and 68.2% in the study-specific index score. Corresponding university-group values were 5.2%, 8.7%, 7.3%, and 39.0%. VIFs did not indicate problematic collinearity (maximum = 2.32 and 1.41). Breusch–Pagan tests indicated heteroscedasticity in both index-score equations (p = .004 and p <.001), motivating HC3 coefficient tests; nonparametric case bootstrap intervals were retained for indirect associations. Maximum Cook’s distances were .144 and .069. Institution-level clustering could not be estimated because school identifiers were not retained and the total number of institutions was small (three senior high schools and four universities). A *post hoc* coefficient-level sensitivity calculation indicated 80% power to detect partial correlations of approximately .237 in the senior-high-school group and .167 in the university group with seven explanatory variables. These values do not constitute power calculations for indirect associations; the bootstrap interval widths remain the primary information about their precision.

## 4 Discussion

We examined adjusted associations and statistical indirect associations in two student groups. Emotional distress accounted statistically for part of the association between parental warmth and index scores in both groups, whereas self-control did so only in the senior-high-school group. The models did not detect an indirect association through escape motivation, even though escape motivation had positive adjusted coefficients. These findings show that a variable can have different statistical roles depending on whether the question concerns its association with the index score or its contribution to the warmth–index-score indirect structure.

### 4.1 Adjusted association does not imply statistical indirect association

Escape motivation played different statistical roles depending on the coefficient examined. In university students, it had a moderate zero-order correlation with the index score and a positive adjusted b path, but the parental-warmth–escape a path was small and positive. The resulting indirect association therefore did not have the negative sign expected for an inverse sequence. In senior-high-school students, the zero-order escape–index-score association was r = .04 and became positive only after adjustment. This change is consistent with suppression or a conditional association and does not support treating escape motivation as a simple independent correlate in this group.

Neither confidence interval establishes that the escape indirect association is absent. Thus, the primary analysis did not detect a statistically significant indirect association through escape motivation.

The MOGQ escape subscale measures gaming used to disengage from unpleasant states. It does not separate brief relaxation from persistent avoidant coping. Prior work indicates that escapism can serve different functions and show different relations with mental health (32). The small positive warmth–escape coefficient may reflect this heterogeneity, but the present study did not test that explanation.

### 4.2 Emotional distress and self-control as candidate variables for prospective study

Parental warmth was associated with lower emotional distress, which was associated with lower index scores in both groups. In the senior-high-school group, warmth was also associated with higher self-control, which was associated with lower index scores. These patterns are compatible with affective and self-regulatory accounts of problematic gaming. Emotional distress and self-control therefore merit prospective investigation as candidate explanatory variables, but the present results do not rank them as established mechanisms or intervention targets.

Longitudinal findings do not point to a single direction. Adolescent IGD has predicted later psychosocial difficulties in some studies (33), while other cohorts report reciprocal links between problematic gaming and depression or loneliness (34), and IGD has also preceded declines in self-esteem and social support among older adolescents and emerging adults (35). Together, these studies suggest that the temporal ordering among IGD, distress, and psychosocial functioning may vary across contexts and should be examined prospectively.

### 4.3 Interpreting the group-specific patterns

The self-control indirect association was detected in the senior-high-school group but not in the university group. This should not be interpreted as evidence that the association was stronger in senior-high-school students, because the item-level self-control model did not support metric invariance. Moreover, the groups differed in educational and recruitment contexts. We therefore restrict interpretation to the within-group patterns.

### 4.4 Implications

Associations with this study-specific index are not sufficient evidence that a variable should be targeted in treatment. Prospective and intervention studies using validated gaming-disorder measures are needed to examine whether changes in distress, self-control, or escape motivation precede and account for changes in the outcomes those measures assess. The present data do not explain the durability of existing treatments, and treatment evidence should be interpreted cautiously because trials have varied in design quality and reporting (36).

### 4.5 Limitations

Several limitations qualify the findings. First, all variables were measured once, so the indirect associations are associational decompositions rather than evidence of temporal mediation (28); shared-method variance may also have contributed because all constructs were self-reported at one occasion (37). Second, educational stage is confounded with school type, recruitment network, and other contextual differences, preventing a clean developmental inference. Institution identifiers were not retained, so clustering could not be modeled. Third, the primary sample included respondents who selected ‘I do not play’; a sensitivity analysis excluding them reproduced the primary inferential pattern (**Supplementary Table S3**). Fourth, the exclusion of 80 respondents was supported by converging response-quality indicators (**Supplementary Table S6**), but sensitivity analyses showed that two small university-group estimates were affected by this screening decision (**Supplementary Table S4**).

Fifth, the four-item distress composite combines brief depression and anxiety screens and should be interpreted as general negative affect rather than a diagnostic construct; the university-group RMSEA was elevated in a two-degree-of-freedom model. Sixth, item-level analyses did not support a unidimensional, metrically invariant self-control measure; therefore, coefficient magnitudes were not compared across groups, and within-group self-control associations also warrant caution. Seventh, the 11-item study-specific gaming-related symptom index has not undergone independent validation. University-group factor-model fit remained imperfect. The added duration-association and scoring-sensitivity analyses are *post hoc*, same-sample checks; they neither establish diagnostic validity nor substitute for independent validation. The regression and indirect-association findings therefore refer to scores on this study-specific index rather than established IGD symptom severity. Eighth, the escape-motivation subscale may not distinguish flexible, temporary disengagement from rigid avoidance. Ninth, the senior-high-school sample (n = 145) provides limited precision for small indirect associations and complex latent models; power for indirect effects and SEM depends strongly on the component paths and model properties (38, 39). Finally, self-report, single-occasion measurement, and recruitment from one Chinese province limit generalizability. Clinical applications should not be inferred from these findings and require longitudinal verification.

## 5 Conclusions

Emotional distress statistically accounted for part of the association between parental warmth and scores on the study-specific gaming-related symptom index in both groups, whereas self-control did so in the senior-high-school group. Escape motivation showed positive adjusted coefficients, but no indirect association through escape motivation was detected. These cross-sectional findings concern scores on an unvalidated study-specific measure and should not be generalized to established IGD symptom severity. Replication with independently validated gaming-disorder measures is needed.

## Data Availability

The deidentified data, accompanying codebook, and supplementary analysis code are provided as Supplementary Material with this article. Further inquiries can be directed to the corresponding author.
