# Supplementary material for: Cross-sectional correlates and indirect associations linking parental warmth to scores on a study-specific gaming-related symptom index in senior-high-school and university students
Source: Front Psychiatry. 2026 Sep 29;17:1928745. doi: 10.3389/fpsyt.2026.1928745 (PMC13658984; doi:10.3389/fpsyt.2026.1928745)
Supplement: Supplementary file 1 [file SupplementaryFile1.zip › Supplementary/Supplementary_Material.docx]

**Supplementary Material**

*Cross-Sectional Correlates and Indirect Associations Linking Parental Warmth to Scores on a Study-Specific Gaming-Related Symptom Index in Senior-High-School and University Students*

This file reports gaming-time coding details, the study-specific gaming-related symptom-index rationale and item content, sensitivity analyses, full correlation matrices, response-quality checks, psychometric analyses, measurement-invariance results, additional post hoc scoring-sensitivity checks, and model diagnostics referenced in the article. All analyses are based on the deidentified dataset supplied with the submission.

# **S1. Gaming-Time Item and Coding**

Administered question (Chinese): ‘你平均每天玩游戏多长时间？’ English translation: ‘On average, how long do you play games each day?’ Original options were: (1) 不玩 [I do not play], (2) 1小时以下 [less than 1 hour], (3) 1–2小时, (4) 2–4小时, (5) 4–6小时, and (6) 6小时以上 [6 hours or more]. Respondents selecting ‘I do not play’ were retained as the lowest ordinal gaming-time category, and their recorded responses to the gaming-specific measures were retained rather than recoded to zero. In the final analytic sample, 33 senior-high-school students (22.8%) and 75 university students (26.3%) selected this category.

# S2. Study-Specific Gaming-Related Symptom Index and Adaptation Rationale

The study used an 11-item study-specific gaming-related symptom index, administered in Chinese and substantially adapted from GADIS-A (Paschke et al., 2020; Chen et al., 2023; main manuscript references 23 and 24). The modifications involved substantive item reformulation and supplementation, not a translation-only or minor adaptation. The index has not undergone independent validation and is not treated as an established measure of IGD symptom severity.

The practical considerations were to use a common item set with senior-high-school and university students while reducing wording tied to parental monitoring, and to describe gaming-related difficulties explicitly across family, academic, peer, sleep, and emotional domains. This explanation is provided retrospectively during revision; it is not evidence from a documented scale-development or validation protocol. The original GADIS-A already includes negative consequences and was developed from the ICD-11 framework. These considerations do not establish that the changes were necessary, superior, or sufficient to ensure comparability.

For example, H1 replaces wording about exceeding planned or parent-agreed gaming with a direct statement about inability to control gaming time; this changes the operationalization rather than merely translating it. H1–H6 are grouped as cognitive-behavioral symptoms and H7–H11 as negative consequences for the reported analyses. These content labels do not imply diagnostic specificity. All 11 items are equally weighted in the mean score; no additional domain weighting or diagnostic cut-off is applied.

**Table S2. Administered items of the study-specific gaming-related symptom index.**

| **Domain** | **Item** | **English rendering of administered Chinese item** |
| --- | --- | --- |
| Cognitive-behavioral symptoms | H1 | I cannot control the amount of time I spend gaming. |
| Cognitive-behavioral symptoms | H2 | The amount of time I spend gaming keeps increasing. |
| Cognitive-behavioral symptoms | H3 | I continue gaming even when I know it has negative consequences. |
| Cognitive-behavioral symptoms | H4 | Even when gaming affects other important things, I still give gaming priority. |
| Cognitive-behavioral symptoms | H5 | I cannot control how often I start gaming. |
| Cognitive-behavioral symptoms | H6 | I cannot control the priority that gaming has in my life. |
| Negative consequences | H7 | Because of gaming, my relationship with my family has worsened. |
| Negative consequences | H8 | Because of gaming, my academic performance has declined. |
| Negative consequences | H9 | Because of gaming, my relationships with friends have been affected. |
| Negative consequences | H10 | Because of gaming, the quality of my sleep has worsened. |
| Negative consequences | H11 | Because of gaming, I feel that my emotional state has worsened. |

Note. The English wording represents translations of the administered Chinese items and should not be interpreted as the official GADIS-A wording. Response options range from 1 (never) to 5 (always). H1–H11 correspond to dataset variables igd_1–igd_11. The dataset names igd_1–igd_11 are retained as legacy identifiers; they do not designate a validated IGD measure.

# **S3. Non-Gamer Exclusion Sensitivity Analysis**

**Table S3. Indirect associations after excluding respondents reporting no gaming.**

| **Group** | **n** | **Indirect via** | **a × b** | **95% CI** | **Inference** |
| --- | --- | --- | --- | --- | --- |
| Senior high school | 112 | Escape motivation | .016 | [−.018, .056] | Not detected |
| Senior high school | 112 | Self-control | −.206 | [−.347, −.096] | Detected |
| Senior high school | 112 | Emotional distress | −.118 | [−.221, −.008] | Detected |
| University | 210 | Escape motivation | .012 | [−.021, .047] | Not detected |
| University | 210 | Self-control | −.016 | [−.047, .009] | Not detected |
| University | 210 | Emotional distress | −.055 | [−.101, −.020] | Detected |

Note. Respondents selecting ‘I do not play’ were excluded from this sensitivity analysis. The remaining sample comprised 112 senior-high-school students and 210 university students. The pattern of indirect associations matched the primary analysis.

# **S4. Relaxed Zero-Variance Screen**

**Table S4. Indirect associations with the zero-variance screen relaxed.**

| **Group** | **n** | **Indirect via** | **a × b** | **95% CI** | **Inference** |
| --- | --- | --- | --- | --- | --- |
| Senior high school | 167 | Escape motivation | .037 | [−.003, .090] | Not detected |
| Senior high school | 167 | Self-control | −.166 | [−.268, −.087] | Detected |
| Senior high school | 167 | Emotional distress | −.100 | [−.180, −.039] | Detected |
| University | 342 | Escape motivation | .033 | [.006, .065] | Small positive |
| University | 342 | Self-control | −.016 | [−.036, −.001] | Small negative |
| University | 342 | Emotional distress | −.062 | [−.102, −.026] | Detected |

Note. Seventy-nine of the 80 screened cases were otherwise age/grade eligible (N = 509). Results are described as screen-dependent, not false or spurious.

# **S5. Full Correlation Matrices**

*Table S5a. Senior-high-school group (n = 145).*

| **Variable** | **Warmth** | **Need sat.** | **Escape** | **Self-control** | **Distress** | Index score | **Sex** | **Age** | **Gaming time** |
| --- | --- | --- | --- | --- | --- | --- | --- | --- | --- |
| Warmth | 1.000 | .843 | .146 | .615 | −.437 | −.640 | −.158 | .112 | −.401 |
| Need sat. | .843 | 1.000 | .202 | .729 | −.446 | −.702 | −.080 | .101 | −.449 |
| Escape | .146 | .202 | 1.000 | .385 | .231 | .041 | −.074 | −.222 | −.062 |
| Self-control | .615 | .729 | .385 | 1.000 | −.046 | −.576 | −.072 | −.082 | −.510 |
| Distress | −.437 | −.446 | .231 | −.046 | 1.000 | .423 | .147 | −.295 | .015 |
| Index score | −.640 | −.702 | .041 | −.576 | .423 | 1.000 | −.072 | −.282 | .600 |
| Sex | −.158 | −.080 | −.074 | −.072 | .147 | −.072 | 1.000 | −.063 | −.175 |
| Age | .112 | .101 | −.222 | −.082 | −.295 | −.282 | −.063 | 1.000 | −.121 |
| Gaming time | −.401 | −.449 | −.062 | −.510 | .015 | .600 | −.175 | −.121 | 1.000 |

*Table S5b. University group (n = 285).*

| **Variable** | **Warmth** | **Need sat.** | **Escape** | **Self-control** | **Distress** | Index score | **Sex** | **Age** | **Gaming time** |
| --- | --- | --- | --- | --- | --- | --- | --- | --- | --- |
| Warmth | 1.000 | .641 | .078 | .233 | −.205 | −.162 | −.096 | .110 | −.098 |
| Need sat. | .641 | 1.000 | .036 | .399 | −.249 | −.225 | −.056 | .061 | −.156 |
| Escape | .078 | .036 | 1.000 | −.248 | .160 | .343 | −.143 | .075 | .184 |
| Self-control | .233 | .399 | −.248 | 1.000 | −.466 | −.335 | .075 | .071 | −.197 |
| Distress | −.205 | −.249 | .160 | −.466 | 1.000 | .385 | −.141 | −.075 | .069 |
| Index score | −.162 | −.225 | .343 | −.335 | .385 | 1.000 | −.328 | −.075 | .430 |
| Sex | −.096 | −.056 | −.143 | .075 | −.141 | −.328 | 1.000 | −.084 | −.392 |
| Age | .110 | .061 | .075 | .071 | −.075 | −.075 | −.084 | 1.000 | −.054 |
| Gaming time | −.098 | −.156 | .184 | −.197 | .069 | .430 | −.392 | −.054 | 1.000 |

# **S6. Response-Quality Evidence for the Zero-Variance Group**

**Table S6. Response-quality indicators in the zero-variance and retained groups.**

| **Indicator** | **Zero-variance group** | **Retained group** | **Effect/statistic** |
| --- | --- | --- | --- |
| Completion time, median (s) | 178.5 | 298.0 | Mann–Whitney p < .001; Hedges g = −.47 |
| Longest identical-response run, median | 29 | 11 | g = 3.14 |
| Unique response categories, median | 3 | 5 | g = −1.75 |
| Within-person response variance, median | .62 | 1.48 | g = −.85 |
| Constant warmth responses | More likely | Reference | OR = 4.98 |
| Constant need-satisfaction responses | More likely | Reference | OR = 8.71 |
| Constant escape responses | More likely | Reference | OR = 20.47 |
| Constant index responses | More likely | Reference | OR = 6.27 |
| Constant distress responses | More likely | Reference | OR = 10.10 |

# **S7. Reliability and Factor-Analytic Checks**

**Table S7. Reliability and factor-analytic checks.**

| **Measure/group** | **α** | **ω** | **Loading range** | **CFI** | **TLI** | **RMSEA** | **SRMR** |
| --- | --- | --- | --- | --- | --- | --- | --- |
| Distress / SHS | .944 | .944 | .853–.938 | 1.000 | — | .000 | .009 |
| Distress / university | .924 | .926 | .800–.905 | .985 | — | .151 | .025 |
| Study-specific gaming-related symptom index / SHS | .965 | .970 | .802–.936 | .978 | .972 | .076 | .026 |
| Study-specific gaming-related symptom index / university | .953 | .968 | .786–.944 | .926 | .906 | .145 | .048 |
| Self-control / SHS | .893 | .893 | — | — | — | — | — |
| Self-control / university | .775 | .777 | — | — | — | — | — |

The university distress model had only 2 degrees of freedom; its elevated RMSEA is therefore interpreted alongside the high CFI, low SRMR, strong loadings, and high reliability. The two-factor model of the study-specific gaming-related symptom index fit substantially better than a one-factor alternative in both groups.

# **S8. Item-Level Measurement Invariance**

**Table S8. Item-level measurement invariance.**

| **Measure** | **Configural CFI** | **Configural RMSEA** | **Metric ΔCFI** | **Scalar ΔCFI** | **Decision** |
| --- | --- | --- | --- | --- | --- |
| Parental warmth | .969 | .090 | −.005 | −.002 | Metric/scalar supported |
| Distress | .991 | .086 | −.003 | .000 | Metric/scalar supported |
| Study-specific gaming-related symptom index (two factor) | .943 | .089 | .001 | −.002 | Small ΔCFI; qualified by model fit |
| Self-control (one factor) | .679 | .110 | −.026 | −.009 | Metric not supported |

Note. Small changes in CFI under equality constraints must be interpreted alongside the fit of the underlying factor model. In particular, university-group index fit was imperfect (S7). These results do not establish diagnostic validity, equivalence with GADIS-A, or unqualified cross-group measurement comparability. The self-control configural and metric results do not support formal comparisons of its coefficient magnitudes.

# **S9. Explained Variance and Diagnostics**

**Table S9. Explained variance and model diagnostics.**

| **Group** | **R² escape** | **R² self-control** | **R² distress** | R² index score | **BP p** | **Max Cook's D** | **Max VIF** |
| --- | --- | --- | --- | --- | --- | --- | --- |
| Senior high school | .085 | .496 | .291 | .682 | .004 | .144 | 2.320 |
| University | .052 | .087 | .073 | .390 | < .001 | .069 | 1.410 |

Institution-level clustering could not be estimated because school identifiers were not retained and only three senior high schools and four universities contributed participants. Coefficient-level 80% sensitivity benchmarks were partial r ≈ .237 (SHS) and .167 (university) with seven predictors; these are not indirect-effect power calculations.

# **S10. Additional Measurement and Scoring-Sensitivity Analyses**

These analyses were undertaken post hoc during revision. They use the unchanged final analytic sample (N = 430; 145 senior-high-school students and 285 university students), inclusion flag, raw item responses, and scoring rules. They supplement, rather than replace, the previously reported factor and invariance results in S7–S8. No new scale-validation sample, diagnostic assessment, or formal between-group contrast was introduced.

## **S10.1. Item-level and score diagnostics**

Corrected item–total correlations are Pearson correlations between each item and the sum of the other ten items, calculated separately within each group. The two domain means retain the item memberships already specified in S2. Neither high reliability nor a large item–total correlation establishes validity.

**Table S10a. Item diagnostics for the administered 11-item index.**

| **Item** | **Content domain** | **SHS: M (SD)** | **SHS: corrected r** | **University: M (SD)** | **University: corrected r** |
| --- | --- | --- | --- | --- | --- |
| H1 | Cognitive-behavioral | 2.15 (1.22) | .845 | 2.21 (1.03) | .746 |
| H2 | Cognitive-behavioral | 2.11 (1.20) | .825 | 2.01 (0.95) | .839 |
| H3 | Cognitive-behavioral | 2.21 (1.30) | .846 | 2.08 (1.06) | .847 |
| H4 | Cognitive-behavioral | 2.07 (1.25) | .840 | 1.84 (0.94) | .863 |
| H5 | Cognitive-behavioral | 2.01 (1.22) | .895 | 1.87 (0.93) | .841 |
| H6 | Cognitive-behavioral | 2.01 (1.24) | .868 | 1.80 (0.92) | .847 |
| H7 | Negative consequences | 1.85 (1.07) | .761 | 1.50 (0.74) | .789 |
| H8 | Negative consequences | 1.99 (1.24) | .839 | 1.69 (0.89) | .783 |
| H9 | Negative consequences | 1.74 (1.12) | .765 | 1.46 (0.71) | .721 |
| H10 | Negative consequences | 2.00 (1.24) | .807 | 1.71 (0.95) | .693 |
| H11 | Negative consequences | 1.89 (1.21) | .813 | 1.60 (0.87) | .704 |

Note. SHS = senior high school. Corrected item–total correlations ranged from .761 to .895 in SHS and .693 to .863 in university students. Cronbach’s α for the full index, H1–H6, and H7–H11 was .965, .957, and .924 in SHS, and .953, .959, and .925 in university students, respectively. These domain scores are sensitivity outcomes, not newly validated subscales.

## **S10.2. Associations with self-reported gaming duration**

Pearson correlations used the six-level ordinal duration code without converting categories into hours. Spearman correlations used average ranks for ties. The same associations were also examined after excluding duration category 1 (the “I do not play” option in S1). Confidence intervals are paired case-resampled 95% percentile intervals based on 5,000 draws within each group and sample. They are descriptive, unadjusted intervals; no multiplicity correction or diagnostic threshold was applied.

**Table S10b. Associations of the full index and domain scores with gaming-duration category.**

**Panel A. Final analytic sample**

| **Group** | **n** | **Score** | **Pearson r** | **95% CI** | **Spearman ρ** | **95% CI** |
| --- | --- | --- | --- | --- | --- | --- |
| SHS | 145 | H1–H11 | .600 | [.459, .714] | .510 | [.359, .640] |
| SHS | 145 | H1–H6 | .645 | [.517, .747] | .569 | [.425, .689] |
| SHS | 145 | H7–H11 | .489 | [.322, .624] | .367 | [.203, .516] |
| University | 285 | H1–H11 | .430 | [.318, .536] | .479 | [.377, .572] |
| University | 285 | H1–H6 | .443 | [.337, .545] | .460 | [.359, .555] |
| University | 285 | H7–H11 | .328 | [.205, .447] | .386 | [.275, .486] |

**Panel B. Excluding respondents reporting no gaming**

| **Group** | **n** | **Score** | **Pearson r** | **95% CI** | **Spearman ρ** | **95% CI** |
| --- | --- | --- | --- | --- | --- | --- |
| SHS | 112 | H1–H11 | .596 | [.445, .727] | .506 | [.341, .649] |
| SHS | 112 | H1–H6 | .636 | [.491, .762] | .583 | [.431, .718] |
| SHS | 112 | H7–H11 | .489 | [.320, .636] | .345 | [.156, .515] |
| University | 210 | H1–H11 | .335 | [.199, .465] | .324 | [.197, .443] |
| University | 210 | H1–H6 | .324 | [.178, .457] | .296 | [.162, .421] |
| University | 210 | H7–H11 | .274 | [.135, .414] | .269 | [.139, .397] |

Interpretation. These are same-survey behavioral associations, not evidence of diagnostic accuracy or independent criterion validation. Greater gaming duration is not synonymous with gaming disorder. H1 and H2 also refer to gaming time, so content overlap may contribute. The positive associations persisted after the no-gaming category was excluded, but neither this pattern nor its magnitude validates the study-specific index.

## **S10.3. Domain-outcome scoring-sensitivity analyses**

The parallel indirect-association model was repeated with the mean of H1–H6 and, separately, the mean of H7–H11 as the outcome. Parental warmth remained the antecedent; escape motivation, self-control, and emotional distress remained simultaneous intervening variables. Sex, within-group-centered age, and ordinal gaming duration were retained as covariates. Each continuous score was standardized using its mean and sample SD across the final N = 430 before group-specific estimation; these scaling constants were held fixed during resampling. No item was selected or removed according to its association with the explanatory variables.

Indirect associations used 5,000 case-resampled percentile-bootstrap draws per group, with the same cases resampled across the two outcome specifications. Table S10c reports all six domain-specific indirect associations per group. The unchanged 11-item reference estimates and their original intervals remain in main Table 3; those point estimates were independently reproduced as a numerical check. No new formal cross-group comparison was calculated.

**Table S10c. Indirect associations under the two alternative domain-outcome specifications.**

**Panel A. Senior-high-school students (n = 145)**

| **Outcome** | **Indirect association via** | **a** | **b** | **a × b** | **95% CI** | **CI excludes 0** |
| --- | --- | --- | --- | --- | --- | --- |
| H1–H6 | Escape motivation | .145 | .172 | .025 | [−.011, .072] | No |
| H1–H6 | Self-control | .592 | −.339 | −.201 | [−.314, −.107] | Yes |
| H1–H6 | Emotional distress | −.555 | .194 | −.108 | [−.176, −.051] | Yes |
| H7–H11 | Escape motivation | .145 | .071 | .010 | [−.018, .040] | No |
| H7–H11 | Self-control | .592 | −.253 | −.150 | [−.273, −.046] | Yes |
| H7–H11 | Emotional distress | −.555 | .293 | −.163 | [−.255, −.089] | Yes |

**Panel B. University students (n = 285)**

| **Outcome** | **Indirect association via** | **a** | **b** | **a × b** | **95% CI** | **CI excludes 0** |
| --- | --- | --- | --- | --- | --- | --- |
| H1–H6 | Escape motivation | .081 | .204 | .016 | [−.009, .046] | No |
| H1–H6 | Self-control | .176 | −.104 | −.018 | [−.045, .004] | No |
| H1–H6 | Emotional distress | −.177 | .276 | −.049 | [−.091, −.016] | Yes |
| H7–H11 | Escape motivation | .081 | .173 | .014 | [−.007, .039] | No |
| H7–H11 | Self-control | .176 | −.028 | −.005 | [−.030, .020] | No |
| H7–H11 | Emotional distress | −.177 | .244 | −.043 | [−.091, −.012] | Yes |

Interpretation. The interval-exclusion pattern matched that of the full index: self-control and emotional-distress indirect associations had intervals below zero in SHS; only the emotional-distress indirect association had intervals below zero in university students. Escape-motivation intervals included zero throughout. This supports robustness to these examined scoring specifications, not equivalence of effect sizes, a true between-group difference, or validation of either domain. Both outcomes are subsets of the same study-specific item set and may share its measurement limitations.

## **S10.4. Reproducibility and scope**

All S10 numerical results were generated from the supplied deidentified CSV using the accompanying S10_Additional_Analyses.py script. The source contains 551 returned questionnaires; the existing included_final = 1 flag selects the unchanged N = 430. No raw values, inclusion flags, or primary manuscript coefficients were overwritten. The added analyses used Python 3.13.5, NumPy 2.3.5, pandas 2.2.3, SciPy 1.17.0, and statsmodels 0.14.6. Their software environment is distinguished from the previously reported environment for the primary analyses.

The master random seed was 20260909. NumPy SeedSequence.spawn(4) generated separate streams for the indirect-association bootstrap in SHS and university students and for the duration-correlation bootstrap in those groups. Percentile endpoints were the 2.5th and 97.5th empirical percentiles. Complete unrounded results, the source-file SHA-256 digest, and execution details accompany the script. Re-running a bootstrap with different random draws can change interval endpoints slightly; S10 does not replace the original primary intervals in Table 3.

The raw questionnaire responses provide no independent clinical diagnosis, standard GADIS-A comparator, or separate validation cohort for these added checks. Therefore, the analyses cannot establish diagnostic accuracy, equivalence with the original GADIS-A, or absence of measurement bias. High item consistency and stable associations across two domain means also do not resolve the imperfect university-group factor fit reported in S7. The original factor-analytic and invariance numbers are retained rather than represented as newly estimated in S10.

Source-file SHA-256: 43e0bd516f7febb1fa5957135f68d28e1281f8480c2035beb67c2728c9c76c04
